# Supplementary material for: Establishment of a Potential Serum Biomarker Panel for the Diagnosis and Prognosis of Cholangiocarcinoma Using Decision Tree Algorithms
Source: Diagnostics (Basel). 2021 Mar 25;11(4):589. doi: 10.3390/diagnostics11040589 (PMC8064492; doi:10.3390/diagnostics11040589)
Supplement: Supplementary file 1 [file diagnostics-11-00589-s001.pdf]

Article

Establishment of a potential serum biomarker panel for the diagnosis and prognosis of cholangiocarcinoma using decision tree algorithms

Phongsaran Kimawaha [1,7], Apinya Jusakul [2,7], Prem Junsawang [4,7], Raynoo Thanan [5,7], Attapol Titapun [6,7], Narong Khuntikeo [6,7] and Anchalee Techasen [3,7*]

Supplementary

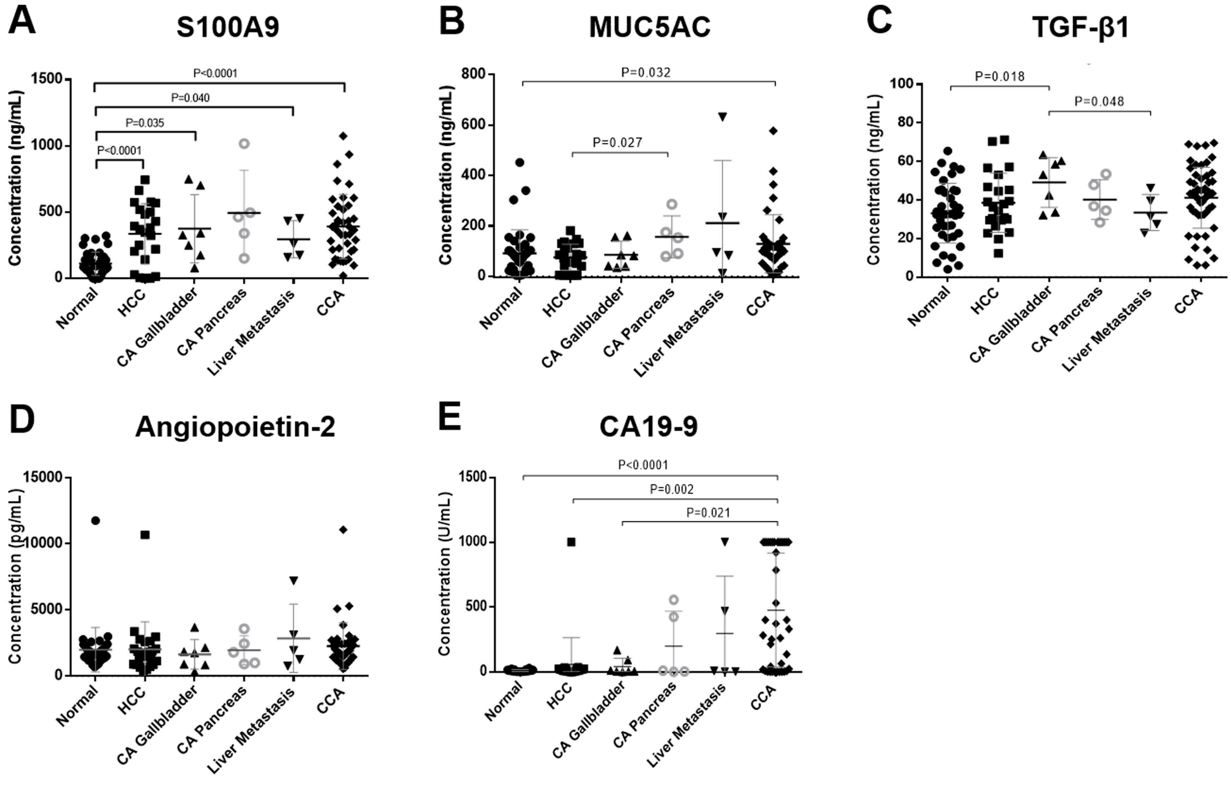

Figure S1. Serum levels of S100A9 (A), MUC5AC (B), TGF-β1 (C), angiopoietin-2 (D), and CA19-9 (E) in normal control group, Non-CCA group, and CCA patients. Non-CCA group including hepatocellular carcinoma (HCC), CA gallbladder, CA pancreas, and liver metastasis patients. Scatter plots represent mean ± standard deviation (SD). The P value < 0. 05 was considered statistically significant when compared in each group.

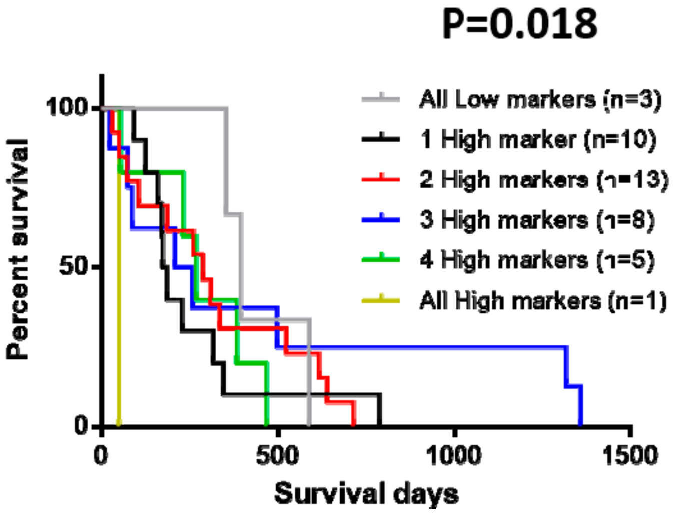

Figure S2. Overall survival analysis according to Kaplan-Meier method with a log rank test calculated for combined biomarkers with survival rate in CCA patients. The P value < 0.05 was considered statistically significant.

Table S1. List of tree's parameters with the sets of their candidate values.

parameter	value	descriptions
max_depth	{3,4,5,6,7,8,9}	The maximum depth of the tree.
max_feature	{1,2,3,4,5}	The number of variables or attributes to consider when looking for the best split.
min_samples_leaf	{3,4,5,…,10}	The minimum number of samples required to be at a leaf node.
min_samples_split	{10,11,13…, ,20}	The minimum number of samples required to split an internal node.
criterion	{'gini', 'entropy'}	The function to measure the quality of a split. Supported criteria are "gini" for the Gini impurity and "entropy" for the information gain.

Table S2. The characteristics of CCA patients.

Patient ID	Sex	Age	Anatomical subtype	Histological type	Survival Day	Recurrent status	TNM Stage	Lymph node metastasis	Distant metastasis	Metastasis
**CCA_01**	F	56	pCCA	papillary	186	1	3a	0	0	0
**CCA_02**	M	57	pCCA	mucinous cystadenocarcinoma	286	0	0	0	0	0
**CCA_03**	M	67	pCCA	papillary	123	0	2	0	0	0
**CCA_04**	M	66	pCCA	tubular	85	0	3b	1	0	1
**CCA_05**	F	63	iCCA	Tubular	229	0	4b	1	1	1
**CCA_06**	M	57	pCCA	tubular	786	1	2	0	0	0
**CCA_07**	M	63	iCCA	Tubular adenocarcinoma	315	1	0	1	0	1
**CCA_08**	M	54	pCCA	papillary	714	1	3b	1	0	1
**CCA_09**	M	58	iCCA	papillary	381	1	4a	1	0	1
**CCA_10**	M	71	pCCA	Tubular adenocarcinoma	71	0	3b	1	0	1
**CCA_11**	F	71	iCCA	tubular adenocarcinoma	334	1	4a	0	0	0
**CCA_12**	M	65	iCCA	tubular	231	1	3a	0	0	0
**CCA_13**	F	69	iCCA	papillary	640	0	4a	1	0	1
**CCA_14**	M	66	iCCA	Adenosquamous types	50	0	4a	1	0	1
**CCA_15**	M	54	iCCA	Cribbiform adenocarcinoma	23	0	4b	1	1	1
**CCA_16**	M	61	iCCA	tubular	73	0	4b	0	1	1
**CCA_17**	F	67	iCCA	intraductal mucinous adenoma	618	0	1	0	0	0
**CCA_18**	M	74	pCCA	tubular adenocarcinoma	523	0	2	0	0	0
**CCA_19**	M	51	iCCA	adenosquamous carcinoma	187	0	3a	0	0	0
**CCA_20**	M	79	dCCA	ND	168	0	4a	0	0	0
**CCA_21**	F	63	pCCA	tubular	53	0	0	1	1	1
**CCA_22**	F	53	pCCA	tubulopapillary adenocarcinoma	467	0	3b	1	0	1
**CCA_23**	M	70	iCCA	tubulopapillary adenocarcinoma	260	0	4a	1	0	1
**CCA_24**	F	69	iCCA	papillary adenoma	587	0	0	0	0	0
**CCA_25**	F	59	iCCA	papillary carcinoma	351	0	4a	1	0	1
**CCA_26**	F	67	iCCA	tubular	170	1	4b	1	1	1
**CCA_27**	M	73	iCCA	tubular	209	0	4a	1	0	1
**CCA_28**	M	43	iCCA	tubular	90	0	4b	1	1	1
**CCA_29**	F	65	iCCA	tubular	497	1	4a	1	0	1
**CCA_30**	M	54	iCCA	tubular	309	1	4a	1	0	1

**CCA_31**	F	44	iCCA	invasive-papillary	344	1	4a	1	0	1
**CCA_32**	M	39	pCCA	papillary	1359	1	2	0	0	0
**CCA_33**	M	56	iCCA	tubular	157	1	4a	0	0	0
**CCA_34**	M	57	iCCA	papillary	267	1	4a	0	0	0
**CCA_35**	M	82	iCCA	tubular	30	0	4a	0	0	0
**CCA_36**	M	52	iCCA	invasive-papillary	49	0	4a	1	0	1
**CCA_37**	F	60	iCCA	invasive-papillary	257	1	4a	1	0	1
**CCA_38**	M	52	iCCA	tubular	104	0	4a	1	0	1
**CCA_39**	M	61	pCCA	tubular	1318	1	3a	0	0	0
**CCA_40**	M	63	iCCA	tubular	396	1	3a	0	0	0
						0 = no		0 = no	0 = no	0 = no
						1 = yes		1 = yes	1 = yes	1 = yes

Abbreviations: intrahepatic CCA (iCCA), perihilar CCA (pCCA), and distal CCA (dCCA).

Table S3. Predictive values of serum TGF-β1 and angiopoietin-2 levels for prognosis metastasis and TNM stages in CCA patients, based on the optimal cut-off derived from ROC analysis and YI calculation.

Group comparisons	AUC (95% CI)	Cut-off	YI	SN	SP	*P* value
**TGF-β1**		**ng/mL**				
No metastasis vs. Metastasis	0.700 (0.557-0.842)	>48.8	0.35	44	91	**0.012**
Early TNM vs. Late TNM	0.748 (0.610-0.885)	>43.6	0.42	51	91	**0.012**
**Angiopoietin-2**		**pg/mL**				
Early TNM vs. Late TNM	0.758 (0.555-0.962)	>1457	0.59	81	78	**0.020**
**TGF-β1 (43.6 ng/mL) + Angiopoietin-2 (1457 pg/mL)**						
Early TNM vs. Late TNM	0.842 (0.704-0.980)		0.32	32	100	**0.002**

AUC = area under the ROC curve, YI = Youden index, SN = sensitivity, SP = specificity

The *P* value < 0.05 was considered statistically significant.

Table S4. Predictive risk of metastasis and TNM stages relative to using serum levels of TGF-β1 and angiopoietin-2.

Comparative diagnosis	OR crude	95% CI (*P* value)	OR* adjust	95% CI (*P* value)
**No metastasis vs. Metastasis**				
TGF-β1 < 48.8 ng/mL vs. ≥ 48.8 ng/mL	5.00	1.24-20.14	5.71	1.31-24.82
		**(0.024)**		**(0.020)**
**Early TNM vs. Late TNM**				
TGF-β1 < 43.6 ng/mL vs. ≥ 43.6 ng/mL	6.86	1.36-34.71	7.73	1.41-42.28
		**(0.020)**		**(0.018)**
Angiopoietin-2 < 1457 pg/mL vs. ≥ 1457 pg/mL	14.58	2.40-88.80	23.22	2.89-186.71
		**(0.004)**		**(0.003)**
TGF-β1 + Angiopoietin-2	14.58	2.40-88.80	23.22	2.89-186.71
		**(0.004)**		**(0.003)**

Abbreviations; OR: odds ratio, CI: confidence interval

*Odds ratio adjusted for age and sex statistical analysis.
